# New Appliances and Things Medical

**Published:** 1906-08-25

**Authors:** 


### NEW APPLIANCES AND THINGS MEDICAL.

### THE AUSTRALIAN WINES AND BRANDY.

(Hans Irvine and Co., Dowgate Hill, Cannon Street, London, E.C.)

We have received samples of the various wines supplied by this firm, and commented upon them in a previous issue. Among the white wines is one to which the name Muscatene (not Muscatel) has been given. It is a sweet wine having a very strongly developed Muscat taste. This company is prepared to send some of their various wines or brandy to any medical practitioner or nurse who is interested in this enterprise free of cost.

### HALL'S WINE.

(Stephen Smith and Co., Ltd., Bow, London, E.)

We have received a sample of this well-known tonic wine. Hall's wine has a well-merited, world-wide reputation as a stimulating and tonic beverage. These virtues it owes to the fact that its proprietors are careful to use for its preparation good wine, and to add to it the extract of good cocoa leaves. This latter constituent makes itself felt not only by the wine's content in its active principle, namely, cocaine, but also by the appetising and refreshing aroma, the existence of which depends upon the constituents of the leaves other than cocaine. The alcoholic strength of the wine is approximately that of good Madeira, and to this is added a considerable percentage of the alkaloid cocaine, in fact quite sufficient to give in half a wine-glassful of the wine an adequate dose of cocaine. As has been pointed out above cocoa leaves contain not only the alkaloid cocaine, but also a substance known as hygrin, and this we regard from the point of view of a general tonic as an advantage. A wine-glassful of this wine corresponds roughly to one drachm of cocoa leaves. The average content of cocoa leaves in cocaine is 4 per cent., hence it would follow that in a wine-glassful, or two ounces of this wine, there would be approximately a fifth of a grain of the alkaloid. The restorative properties of this wine are well known, and we think it is a preparation highly to be recommended.

### ASEPTIC PRODUCTS.

(Fassett and Johnson, 31 and 32 Snow Hill, London, E.C.)

We have received samples of some very interesting appliances for surgical purposes which cannot fail to be particularly useful to the general practitioner, especially in country districts. Messrs. Seabury and Johnson's products from the pharmacal laboratories of New York are prepared with a full knowledge of modern requirements, and most ingenious devices have been adopted to secure the absence of contamination for the medicated bandages which they are desirous of submitting to the profession. The preparations are placed in air-tight containers so that when submitted to the most stringent tests the sterilised gauze, lint, bandage, or wool, comes out of the cartons in which they are packed in the factory absolutely germ free and can be used in the most delicate and dangerous operations with perfect safety. Mead's moist gauze, borated 10 per cent., besides being carefully cartoned, is supplied in an actinic glass bottle with a screw-stopper carefully sealed with a paper label to prevent interference with the contents. Over and above that, around the glass cover is screwed a metallic close-fitting rim, effectually excluding any air or germs and maintaining the gauze in a state of absolute sterility. The same may be said of the manner in which the iodoform 5 per cent. absorbent gauze has been preserved from contamination. A highly glazed carton, sealed all round and varnished, prevents the access of air or any interference, and when opened, the gauze will be found to be in a state safe and trustworthy and suitable for any dressing where iodoform is desired. Another bandage which should prove of great service to practitioners, handily put up, easily opened, and thoroughly reliable as regards its contents, is the Plaster of Paris bandage prepared by this firm. Mead's adhesive rubber, neatly put up on rollers is in strict harmony with regard to cleanliness and reliability with the other products of this firm. Another production of this firm is cat-gut sterilised at 330° F. and packed under every antiseptic condition. Thirty inches of catgut are placed in a sterilised, aseptic, sealed, germ-proof capsule. Seabury's Chromic Catgut No. 1., sterilised, is enclosed in a hermetically sealed glass tube niched at a point so as to be easily fractured when required.

